# On the Removal of Loose Cartilages from the Knee-Joint

**Published:** 1829-01-01

**Authors:** 


### XIII.

### CHELTENHAM CASUALTY HOSPITAL.

I On the Removal of loose Substances from the Knee-joint. By Charles Averill, Esq.

There is probably no disease to which the knee is subject, that produces more excruciating pain, for short periods, than that which is occasioned by cartilaginous or bony substances lying loose in the cavity of the joint. The following observations we shall give in the words of Mr. Averill.

"When it is ascertained that one or more of these substances are lying loose in the cavity of the knee-joint, we have the choice of two modes of practice, which may be called the palliative and the curative. The former is the method proposed by the late Mr. Hey, of wearing a bandage, or laced knee-cap, so as to confine the substances in one spot, and, thereby, prevent its giving pain, by getting between the extremities of the bones forming the joint. This practice, I should imagine, is not applicable to those cases in which there are two or more substances present; especially if they differ considerably in size, and if the patient's occupation subject him to hard labour or severe exertion. In such cases, relief may be afforded by the operation of removing the substances; but this, from its necessarily laying open the joint, as well as from its having been, in some instances, unsuccessfully attempted, has always been considered a serious undertaking.

"The only difficulty that, as far as I am informed, has been found in accomplishing the operation, even when there are two or more substances present, is to fix them, whilst the operator cuts into the joint, so that he may extract them readily, after the incision is made. This difficulty, which is owing to the polished surfaces of the loose bodies, and the lubricating nature of the synovia favouring their slippery passage from one part of the joint to another, obliged the surgeon to relinquish the operation, even after he had cut into the joint, in a case of this kind, which was lately related to me by Mr. Thomas Christie, an apprentice of Doctor Ballingall, Surgeon to the Royal Infirmary, in Edinburgh. In this case, the operation had been twice attempted, by different surgeons, without success; and the patient afterwards went into the Edinburgh Infirmary, where the substance was removed by Mr. Allan.

"Aware of the above facts, I was induced to consider how I might obviate the difficulties I have stated, and have been gratified to find that I could do so by very simple means. When the patient, whose case is here introduced, came under my care, I procured an iron ring, represented in the plate, and found, upon trial, that the loose substances in his knee-joint were to be easily fixed by it, so securely, in one spot, as could leave no doubt in my mind of their being easily extracted. The result will best appear in my notes of the case, which are as follows.

"George Fluck, aged 30, by trade a gardener and nurseryman, was admitted into the Cheltenham Casualty Hospital, August 16, 1825, when he gave the following account of himself.

"He had, for several years, thought there was a degree of weakness in his knees, particularly when he was carrying any heavy weight. Between two and three months since, after he had been kneeling for some time in the garden, at work, he was attacked with considerable inflammation and swelling in the left knee, for which he used an embrocation, and when the swelling went down, he found there was a moveable substance in the joint. Shortly after, he discovered a second. These, at times, caused excruciating pain, more particularly when he was walking down hill, or coming down stairs, so as to oblige him to sit down till the pain had subsided.

"He had worn a bandage, by means of which he could fix the larger substance at the upper and outer part of the joint; but the smaller one could not be retained in any one place, and it was this, which, from its motion, and from its getting between the ends of the bones, gave him pain.

"At the time of his admission, both substances could be readily felt, and moved to different parts of the joint; one appeared to be about the size of a marble, flattened; the other considerably smaller.

"He was recommended to submit to the operation of having them removed, to which he consented; and was therefore directed, by way of preparation, to take some pills of calomel and extract of colocynth, and some aperient medicine by day, for two or three days, and to eat no meat.

"On the 19th, the operation was performed in the following manner.

"Both the substances being pushed to the upper and outer side of the joint, and the integuments drawn tightly over them towards one side,* while the knee was kept straightened; the substances were fixed by means of the ring, which I held with my left hand, firmly pressed against the side of the outer condyle of the femur, thus rendering their escape back into the joint impossible.

"I then, with a common scalpel, made an incision, within the ring, through the integuments and capsular ligament, from above, downwards into the joint; when the larger substance immediately fell out on the floor, and, with my finger, I tilted out the smaller one.

"The operation was performed in less than a minute, and only about a drachm of synovia escaped. There was no bleeding of consequence; the lips of the wound were brought together by adhesive plaster, a bandage applied, and a long splint was fixed on the outside of the limb, to prevent the knee being bent. He was directed to keep quiet in bed, and to take a saline draught every three hours.

"August 20.—He has had a good night, and is free from pain.

"22.—The wound dressed, looking very healthy.

"28.—Sat up for an hour or two.

"Sept. 3.—Discharged quite well.

"In conclusion, I may be allowed to ask, whether the evils so much dreaded in the operation of removing loose cartilages from the joints, may not, in all probability, have arisen from the excessive escape of synovia, and the irritation produced by unsuccessful attempts to squeeze out those substances at a wound made comparatively upon speculation; and whether, if they can be always certainly and securely fixed, by the simple means I have employed, the operation be not thereby rendered sufficiently safe to authorise us to recommend it with confidence; at all events, where the bandage and knee-cap have failed to afford relief."—*Midland Reporter, No.* 1.

* "This was done to prevent the wound in the integuments being parallel to that in the capsular ligament."

